# Candida Carriage Rate and Growth Characteristics of Saliva in Diabetes Mellitus Patients: A Case-Control Study

**DOI:** 10.15171/joddd.2015.048

**Published:** 2015-12-30

**Authors:** Preethi Balan, Subhas B Gogineni, Sucheta Kumari N, Veena Shetty, Anusha Lakshman Rangare, Renita L Castelino, Fazil Areekat K

**Affiliations:** ^1^Senior Lecturer, Department of Oral Medicine and Radiology, Faculty of Dentistry, Sree Anjaneya Institute of Dental Sciences, KUHS University, Calicut, India; ^2^Professor and HOD, Department of Oral Medicine and Radiology, Faculty of Dentistry, AB Shetty Memorial Institute of Dental Sciences. Nitte University, Mangalore, India; ^3^Professor, Department of Biochemistry, K.S. Hegde Medical Academy, Nitte University, Mangalore, India; ^4^Professor, Department of Microbiology, K.S. Hegde Medical Academy, Nitte University, Mangalore, India; ^5^Department of Oral medicine and Radiology, Century International Institute of dental Sciences & Research Center, KUHS University, Kasargode, India; ^6^Department of Oral medicine and Radiology, AB Shetty Memorial Institute of Dental Sciences, Nitte University, Mangalore, India; ^7^Department of Oral medicine and Radiology, Sree Anjaneya Institute of Dental Sciences, KUHS University, Calicut, India

**Keywords:** Diabetes mellitus, Candida albicans, glucose

## Abstract

***Background and aims.*** The aim of this study was to establish a relationship between salivary glucose levels and *Candida* carriage rate in type 2 diabetes mellitus patients and assess the growth characteristics and acid production of *Candida* in glucose-supplemented saliva.

***Materials and methods***. A total of 90 subjects, 30 with controlled type 2 diabetes, 30 with uncontrolled type 2 diabetes and 30 without diabetes (control subjects), aged 30‒60 years, participated in the study. Unstimulated saliva was collected and investigated for glucose levels (GOD-POD method), colony-forming units (CFU) of *Candida * and salivary pH, using Indikrom paper strips). Analysis of statistical significance of salivary glucose and PH levels was carried out using post hoc Tukey HSD test. Correlation of *Candida* carriage rate with salivary glucose and salivary PH in the study groups and control group was made using Pearson’s correlation.

***Results.****Candida* CFUs were significantly higher in diabetic subjects, with a significant and positive correlation with salivary glucose levels. There was a negative correlation between salivary PH levels and *Candida* carriage rate.

***Conclusion.*** Increased salivary glucose was associated with increased prevalence of oral *Candida* in diabetic subjects. The growth of *Candida* in saliva was accompanied by a rapid decline in PH, which in turn favored their growth.

## Introduction

Diabetes mellitus is a complex multisystem disorder representing one of the major chronic health problems the world is facing today. The prevalence among adults aged 20-70 years is expected to rise from 285 million in 2010 to 438 million by the year 2030.^1^

Oral candidiasis and other opportunistic fungal infections are some of the early, non-specific signs of uncontrolled diabetes.^2^The carriage of *Candida* species and the density of *Candida* growth in the oral cavity is frequently claimed to increase in patients with diabetes mellitus contributing to acquiring oral candidiasis in such patients.^3,4^*Candida* is known to be a normal commensal of the oral cavity. But during hyperglycemic episodes, the environmental alteration in the oral cavity such as immune dysfunction, increased salivary glucose and acid production favor the transition of the harmless commensal to a pathogen. Hence, estimating the critical *Candida* carriage rate in saliva at which it transforms to produce pathogenesis, especially when it is omnipresent during commensal as well as pathogenic states, can be a valuable aid in identifying patients with increased risk for the disease before development of the occult disease. Institution of prevention strategies at this stage itself can reverse the impending disease as well as its multiple sequelae. Saliva offers an inexpensive and noninvasive screening method as compared to serum in terms of collection, storage and voluminous sampling. Therefore, the present study was undertaken to establish a relationship between salivary glucose levels and *Candida* carriage rate in type 2 diabetes using saliva as a screening medium. Considering the paucity of available information, this study might lay a foundation for the application of saliva as a tool for predicting candidiasis in the population susceptible to diabetes mellitus.

## Materials and Methods

### Study Setting, Design and Subjects 

This randomized case control study was conducted on the south Indian population during 2011‒2012. The study was approved by the ethics committees of the University. Participants provided written informed consent prior to data collection. The study sample consisted of 90 subjects in the age group of 30 to 60 years and included 31 females and 59 males. They were divided into 3 equal groups of 30 patients— Group I included control patients with Random Non Fasting Plasma Glucose (RNFPG) levels less than 120 mg/dL; Group II included patients with controlled diabetes mellitus (RNFPG levels in the range of 120 mg/dL to 200 mg/dL); Group III consisted of patients with uncontrolled diabetes mellitus (RNFPG levels more than 200 mg/dL). As HbA1c reflects the average blood glucose concentration over an extended period of time and remains unaffected by short-term fluctuations in blood sugar levels,^4^ RNFPG level was employed to achieve direct and simultaneous correlations with saliva glucose concentrations. Patients with a history of any systemic or oral mucosal disease, patients who were on medications other than anti-diabetic drugs and patients with habits like smoking, tobacco or betel nut chewing and alcohol consumption were excluded from the study. To avoid bias in the results, confounding factors which might alter the parameters of saliva like oral mucosal or dental disease and subjects with inadequate oral hygiene were excluded from the study.

### Sample Collection and Laboratory Techniques

Unstimulated saliva was collected after 12 to 16 hours of fasting in the morning using a “spit technique” which represented whole mouth fluid contributed by secretions from major and minor salivary glands and potentially gingival crevicular fluid. Salivary glucose was estimated by GOD-POD method. Saliva sampling for estimation of colony-forming units (CFUs) of *Candida* was performed using the oral rinse technique. The rinse was immediately concentrated by centrifuging at 1,700 rpm for 10 minutes. The supernatant was discarded and 0.1 μL inoculating loop was used to spread the sample onto Sabouraud’s dextrose agar plates supplemented with chloramphenicol (10 mg/mL). Cream-colored opaque colonies with yeasty odor were identified. CFUs were counted manually, and the number was multiplied by 1,000 and expressed as CFU/mL. The salivary pH of the collected saliva was estimated using Indikrom paper strips and reported accordingly.

### Statistical Analysis 

Data was analyzed with SPSS 18. Data obtained was analyzed using one-way ANOVA and chi-squared test for comparison between the groups. Analysis of statistical significance of salivary glucose and pH levels was made using post hoc Tukey test. Correlation of *Candida* carriage rate with salivary glucose and salivary pH in the study groups and control group was made using Pearson’s correlation.

## Results

The mean age in group I was 48.20±7.131 years whereas the mean ages in groups II and III were 48.33±8.46247 and 47.833±7.022 years, respectively. Overall a male predominance was observed in all the 3 groups (Table 1). The mean salivary glucose level in group I was calculated at 1.18±0.675 gm/dL, which was lower than that in group II, i.e. 4.95±6.61 gm/dL. The mean salivary glucose level in group III was 13.35±6.61 gm/dL which was the highest.

**Table 1:** Age and gender distribution

**Groups**	**Gender distribution frequency (Percent)**	**Mean age**	**Std. deviation**	**Minimum**	**Maximum**
**Normal (N = 30)**	13 (43.3%) female	48.2000	7.13128	35.00	59.00
	17 (56.7%) male				
**Controlled diabetes mellitus (N = 30)**	5 (16.7%) female	48.3333	8.46222	34.00	59.00
	25 (83.3%) male				
**Uncontrolled diabetes mellitus (N = 30)**	13 (43.3%) female	47.8333	7.02254	35.00	59.00
	17 (56.7%) male				

Statistically significant differences were obtained when salivary glucose levels were compared between group I, II and III (Table 2). The statistical comparison of the salivary pH levels of group I and group II showed no statistically significant differences (P=0.990). Comparison of salivary pH levels between groups II and III showed a significant difference (P=0.002). Similarly, comparison of the salivary pH levels of groups I and III revealed significant differences (P=0.001) (Table 3).

**Table 2:** Analysis of statistical significance of salivary glucose levels using post hoc Tukey tests

**Dependent Variable**	**(I)**	**(J)**	**Mean Difference (I-J)**	**Std. Error**	**Sig.**
**Salivary glucose levels(m/dl)**	Normal	Controlled diabetes mellitus	-3.768	1.057	0.002
		Uncontrolled diabetes mellitus	-12.165	1.057	<0.001
	Controlled diabetes mellitus	Uncontrolled diabetes mellitus	-8.397	1.057	<0.001
^*^DM Diabetes Mellitus					

**Table 3:** Analysis of statistical significance of salivary pH levels using post hoc Tukey tests

**Dependent Variable**	**(I)**	**(J)**	**Mean Difference (I-J)**	**Std. Error**	**Sig.**
**Salivary PH**	Normal	Controlled diabetes mellitus	0.033	0.253	0.990
		Uncontrolled diabetes mellitus	0.933	0.253	0.001
	Controlled diabetes mellitus	Uncontrolled diabetes mellitus	0.900	0.253	0.002

In group I, 46.7% of patients had no *Candida* growth while 40.0% had a *Candida* count of 10 CFU/mL. No patients were observed in the colony forming range of 10^3^ to 10^4^ CFU/mL. In group II 43.3% of patients had colony forming units of 10^2^ CFU/mL whereas no patients with *Candida* growth of more than 10^3^ CFU/mL were observed. The percentage distribution of *Candida* counts in group III showed that *Candida* growth ranged from 10 to 10^4^ CFU/mL with *Candida* growth predominantly in 10^3^ CFU/mL range*.* None of the patients in Group III were observed with negative *Candida* cultures. Comparison between descriptive statistics of groups I, II and III showed that *Candida* count was highest in group III, followed by groups II and I (Table 4).

**Table 4:** Comparison of *Candida* with groups using chi-squared test

		**Oral Candidal carriage(CFU/ml)**					**Total**
		**0**	**10**	**10** ^ 2 ^	**10** ^ 3 ^	**10** ^ 4 ^	
**Normal**	Count	14	12	4	0	0	30
	%within groups	46.7%	40.0%	13.3%	0.0%	0.0%	100.0%
**Controlled diabetes mellitus**	Count	5	8	13	4	0	30
	%within groups	16.7%	26.7%	43.3%	13.3%	0.0%	100.0%
**Uncontrolled diabetes mellitus**	Count	0	8	9	7	6	30
	%within groups	0.0%	26.7%	30.0%	23.3%	20.0%	100.0%
**Total**	Count	19	28	26	11	6	90
	%within groups	21.1%	31.1%	28.9%	12.2%	6.7%	100.0%
^*^CFU Colony Forming Units							

**Figure 1.**
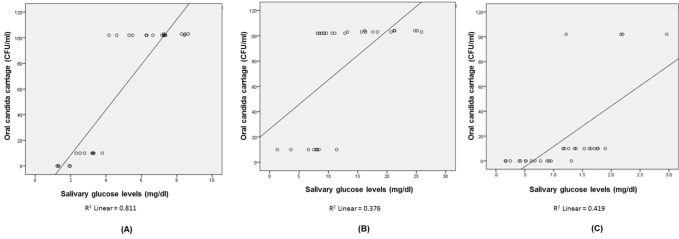
Correlation between salivary glucose and candidal carriage in study groups and control group.

Excellent correlation was observed between salivary glucose levels and *Candida* carriage rates in the three groups, with statistically significant p-value of <0.001 and r-values of 0.844, 0.925, 0.901, respectively. The correlation was seen in 71.2336% of patients in group I, 85.5625% in group II and 81.1801% in group III (Figure 1).

**Figure 2.**
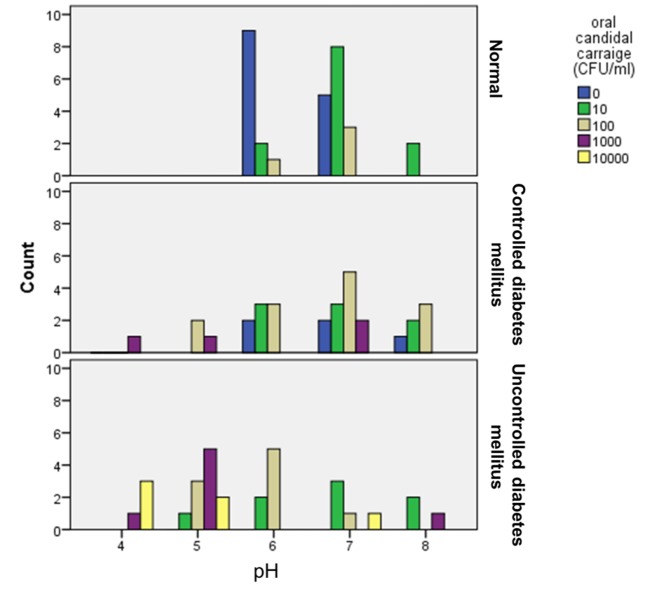
Correlation between salivary pH and candidal carriage in study groups and control group.

A negative correlation was noted between salivary pH and *Candida* carriage rates in patients with uncontrolled diabetes with 39.5641% of cases showing the correlation with an r-value of -0.629 while the correlation observed in group II was poor with only 3.385% of cases showing correlation with an r-value of -0.184 (Figure 2).

## Discussion

The present microbiological study comprehensively investigated oral yeast colonization; candidal CFUs were significantly higher in diabetic subjects (group III > group II) when compared with non-diabetic subjects (group I). This is consistent with the findings of earlier studies by Safia et al (2010),^6^ Fisher et al (1987),^7^ Epstein et al (1980)^8^ and Sashikumar et al (2010).^4^ However, the carriage rate of *Candida* in the oral cavity was different in various studies, which might be attributed to the different sampling methods (such as swabs, water and buffer etc) used by different workers. The oral rinse technique with phosphate-buffered saline was used for sampling in the present study since this is known to be a sensitive technique for estimating the oral candidal carriage.^9^ Moreover, as *C. albicans* is not uniformly distributed in the oral cavities of healthy or diabetic patients, the oral rinse technique can be used where quantitative and overall carriage of yeasts are to be assessed (as in the present study) whereas swabs can be used for examination of specific sites for carriage.^10^

In the present study there was a significant and positive correlation between salivary glucose and CFU of *Candida*, which was in accordance with previous studies where increased *Candida* reflects increased salivary glucose levels.^4,11^

Normal glucose levels in the saliva do not significantly affect oral health or support the growth of microorganisms. However, high levels of salivary glucose increase candidal adherence to buccal epithelial cells.^12^ Salivary glucose forms chemically reversible glycosylation products with proteins in tissues during hyperglycemic episodes, and this leads to accumulation of glycosylation products on buccal epithelial cells, which in turn may increase the number of available receptors for *Candida*.^4^ This enhances their colonization on teeth and oral mucous membranes. Glucose also serves as a nutrient for *Candida* microorganisms and suppresses the killing capacity of neutrophils, which further accentuates colonization and likely consequences can be proposed as a result of these elevated salivary glucose levels in diabetes.^13^

Although the present study confirmed that *Candida* is more prevalent in the oral cavity of diabetics than non-diabetics, some studies have failed to show a correlation between candidal carriage rates and diabetic status.^14,15^Based on a hypothesis diabetes mellitus is not in itself usually responsible for a significant increase in candidal populations, but only when it interacts with a local factor, it can be a possible explanation for these findings.^16^ Khaled Abu-Elteen et al^17^ in their study also observed that the frequency of *Candida* isolation was significantly higher in smokers than in the non-smokers in both diabetics and controls.

*Candida* becomes more easily established in the oral cavities of diabetics than in healthy subjects, but in the dentate diabetic patients, the number of yeasts on the mucosa remains within the normal range as it is limited by masticatory movements, salivary flow and epithelial desquamation.^18^ In the present study, patients with oral mucosal diseases and smoking habits were excluded but dentate and edentulous patients were included in the study indiscriminately, which could be a possible cause for an increase in candidal carriage in these subjects.

In the present study, 53.3% of non-diabetic patients were positive for candidal culture, suggesting candidal carriage even in healthy individuals. But a higher carriage rate was found in diabetic patients as compared to normal controls. The present study also confirmed that the patients with uncontrolled diabetes carried *Candida* more frequently and in higher numbers than those with moderate or good control diabetics. This observation was consistent with previous studies in which a correlation between diabetic controls at any one time and the extent of yeast colonization of oral mucosa sites commonly inhabited by yeasts was established.^14^ The degree of colonization of the mouth by yeasts might be quickly and directly altered by blood glucose levels. However, in a study by LM Tapper Jones et al,^18^ no differences in candidal status could be detected according to the degree of control of diabetes, which is contrary to our study. These contradictory findings may be due to differences in sampling techniques or in dental status and smoking habits between patients.

The total number of yeasts present is important to the development of infection. In order to change from a commensal to a pathogenic mode of life, yeasts need to aggregate in large numbers to accumulate sufficient enzymes for penetration of mucous membranes and epidermis.^14^

Colony forming unit (CFU) is usually recorded to obtain the clinical data, which is essential to establish a clinical diagnosis of oral candidiasis. In the present study, the majority of the patients with controlled diabetes had candidal carriage of 0 to 1000 CFU/mL of saliva while patients with uncontrolled diabetes demonstrated a carriage ranging from 10 to 10,000 CFU/mL of saliva. Negative cultures were obtained in 46.7% of control patients. These observations were consistent with a study by Renner et al,^16^ in which *C. albicans* never exceeded 100 organisms per square centimeter in the control population whereas the concentration always exceeded 10,000 organisms per square centimeter in patients with candidiasis.

However, Epstein et al^8^ demonstrated that carriers and patients with oral candidiasis can be reliably distinguished on the basis of quantitative cultures. Patients with candidiasis had >400 colony-forming units per mL of saliva, whereas carriers of *C. albicans* had <400 colony-forming units per mL. The possible explanation for higher values of CFU/mL in the present study could be differences in colony counting methods used in the two studies. In the present study, CFUs were counted manually, and the number was multiplied by 1,000 and expressed as CFU/mL whereas automated analysis methods were used in the study by Epstein et al.^8^

Thus this observation in the present study requires more comprehensive evaluation with emphasis on laying down cut-off limits for CFU between healthy and diabetics, which might serve as a useful clinical indicator and aid in the diagnosis of oral candidiasis.

A statistically significant difference was seen in salivary pH levels between normal patients and uncontrolled DM patients but statistical comparison of the normal group and group with controlled DM showed no significant differences. The fall in pH in diabetic patients in the present study can be attributed to the decrease in salivary flow rate in diabetic patients and acid production of oral isolates of *Candida* species in the presence of glucose.

In the present study there was a negative correlation between salivary pH levels and *Candida* carriage, consistent with studies carried out by Samaranayake et al,^5^ where the growth of *C. albicans* in human saliva supplemented with glucose was accompanied by acid production. Their study provided the first, detailed qualitative and quantitative data on the short-chain carboxylic acids produced by *Candida* species cultured in human whole saliva. Pyruvates and acetates are, by far, the predominant ionic species that bring about a rapid decrease in pH with both species of *Candida*. The acidic pH conditions prevailing intraorally could, in turn, activate the extracellular phospholipases and acid proteases of *Candida*,^5^ further promoting yeast adhesion to oral mucosal surfaces.^12^ MacFarlane et al^19^ also supported the possible mechanism whereby low pH conditions may promote oral colonization due to the superior ability of yeasts to adhere to epithelia and denture acrylic surfaces at a low pH of approximately 2 to 4.14. Another mechanism may be the aciduric and acidophilic nature of *Candida* species, which allows them to thrive in a low-pH milieu.

Therefore, the present findings taken together would strongly indicate that production of acids in *Candida* species could play a contributory role in the pathogenesis of oral candidiasis.

## Conclusion

In conclusion, the present findings show that the increase in salivary glucose levels in diabetic subjects is likely to contribute to their increased candidal carriage and the potential for increased susceptibility to oral candidiasis. Thus saliva can be used as a reliable noninvasive tool to monitor candidal carriage in diabetic subjects.
